# FRET analysis of the temperature-induced structural changes in human TRPV3

**DOI:** 10.1038/s41598-023-36885-9

**Published:** 2023-06-21

**Authors:** Jinyoung Kim, Jongdae Won, Dong Kyu Chung, Hyung Ho Lee

**Affiliations:** Department of Chemistry, College of Natural Sciences, Seoul National University, Seoul, 08826 Korea

**Keywords:** Biochemistry, Structural biology

## Abstract

Transient receptor potential vanilloid member 3 (TRPV3) is an ion channel that plays a critical role in temperature sensing in skin. There have been active studies on how TRPV3, which is also known as one of the temperature-sensitive transient receptor potential (thermoTRP) channels, responds to temperature. However, the previous studies were mostly based on TRPV3 originating from mice or rats. Here, we focus on human TRPV3 (hTRPV3) and show that which domain of hTRPV3 undergoes conformational changes as temperature increases by Förster resonance energy transfer (FRET) assay. During the heat-induced activation of hTRPV3, the linker domain close to C-terminus, that is, the C-terminal domain shows a largest structural change whereas there is little change in the ankyrin repeat domain (ARD). Interestingly, the activation of hTRPV3 by an agonist shows structural change patterns that are completely different from those observed during activation by heat; we observe structural changes in ARD and S2–S3 linker after ligand stimulation whereas relatively little change is observed when stimulated by heat. Our results provide insight into the thermal activation of hTRPV3 channel.

## Introduction

Temperature sensing is essential for the maintenance of body homeostasis, primarily mediated by specific ion channels, particularly temperature-sensitive transient receptor potential channels (thermoTRPs)^1,2^. ThermoTRPs are activated over a wide range of temperatures, sensing various temperature ranges from noxious cold (< 15 °C) to noxious heat (> 45 °C) depending on the channel type^3^. Among these, transient receptor vanilloid member 3 (TRPV3) is one of the thermoTRPs, activated by warm temperatures ranging from 31 °C to 39 °C^4^.

Recent studies have provided structural information on TRPV3, revealing that each subunit consists of amino-terminal ankyrin repeat domain (ARD), transmembrane domain which is made up of S1–S4 domain and S5-S6 pore domain, linker domain consisting of the pre-S1 and three-stranded β-sheet, and the C terminus^5,6^. These studies determined the structures of mouse TRPV3 (mTRPV3) at 42 °C in both detergent and nanodisc environments, with the activation mechanism by heat appearing consistent across both conditions, indicating that there are two steps in the temperature-dependent activation process; sensitization and channel opening^7,8^. Throughout this activation process, the C terminus, linker domain, S2–S3 linker, and S6 helix undergo structural changes accompanied by the rotation of the intracellular skirt^7,8^. As human proteins have direct relevance to human diseases, research on human TRPV3 (hTRPV3) is essential to lay the foundation for the development of therapeutics. However, due to difficulties such as low expression level and stability of hTRPV3 protein, previous studies are mostly based on the proteins from rodents; therefore, the mechanism by which hTRPV3 senses temperature changes remains unknown^7–9^.

TRPV3 is activated not only by heat but also by various chemicals, including synthesized 2-aminoethoxydiphenyl borate (2-APB) and natural products such as carvacrol and thymol^10,11^. Until now, a structure of mTRPV3 activated by a ligand has been identified only when 2-APB is treated, and there is limited information about the structure when other agonists are applied to human TRPV3^5,6,12,13^. Carvacrol, one of the TRPV3 agonists, acts as a skin allergen and specifically activates TRPV3^11,14^. In a recent paper, it was suggested that the pocket formed by the S2–S3 linker, which is a unique site not involved in 2-APB binding site, might be important for carvacrol binding and channel activation by carvacrol^15^. However, no current structure of the carvacrol-bound TRPV3 is available, impeding the understanding of the carvacrol-gated activation mechanism of TRPV3 and how it differs from the temperature-gated activation mechanism.

In this paper, we focused on hTRPV3, which is a calcium-permeable non-selective ion channel^4^. Heat-induced gating of TRPV3 is hysteretic, meaning that the first heat stimulation is not sufficient to open the channel, and the channel is activated by repeated stimulation^16^. TRPV3, mainly expressed in skin keratinocytes, is crucial for skin physiology by regulating skin barrier formation, hair morphogenesis, hair follicle cycling, wound healing, and inducing cutaneous pain and itch^17,18^. Skin pathology is also related to TRPV3 channels, as verified by experimental results showing that ‘gain-of-function’ mutations in TRPV3 are associated with atopic dermatitis and Olmsted syndrome, and that alterations in TRPV3 expression level cause rosacea and psoriasis^18,19^. To understand how TRPV3 is activated by temperature and is involved in disease at the molecular level, structural studies are essential. We purified full-length hTRPV3 and observed conformational changes in hTRPV3 channel with temperature changes using a Förster resonance energy transfer (FRET) assay. In the FRET assay, remarkable changes were observed in the C-terminal domain when hTRPV3 was stimulated by heat, and this was different from the domain in which the conformational change occurred when stimulated by the agonist, carvacrol. Our results illustrate that hTRPV3 is activated in a similar way to mTRPV3 when exposed to heat, and its mechanism is distinct from the ligand-dependent activation pathway. Moreover, our findings reveal that FRET analysis using purified hTRPV3 enables us to demonstrate both direct heat-induced structural changes and natural compound-induced structural changes, and we propose distinct activation mechanisms for thermal and chemical stimuli.

## Results

### Constructs of human TRPV3 for FRET assay

To generate hTRPV3 constructs for FRET assay, we designed single-cysteine constructs containing only one cysteine in the desired domain. There are 13 cysteine residues in the full-length hTRPV3 sequence, and we generated a cysteine-free (CF) hTRPV3 construct by substituting these native cysteine residues with other amino acids including C75A, C80A, C131A, C146A, C171A, C271A, C446L, C496L, C550F, C612A, C619A, C721A, and C731A. Subsequently, we selected and mutated one residue to cysteine per each construct resulting in constructs containing single cysteine. The selection of residues for labeling was based on several criteria. Firstly, we aimed to use native cysteine residues as much as possible to minimize potential artifacts associated with non-natural cysteine mutation. Secondly, we selected residues that could represent each structural domain of the protein (Supplementary Table 1). Thirdly, we selected residues that were located on the solvent-accessible surface of the protein, to ensure easy access of the labeling reagent to the target residue. Fourthly, we considered the distance between subunits, since FRET can occur when the distance between the donor and acceptor is less than 10 nm. Therefore, we selected residues that were within 10 nm of each other for labeling, in order to facilitate FRET-based measurements.

**Figure 1:**
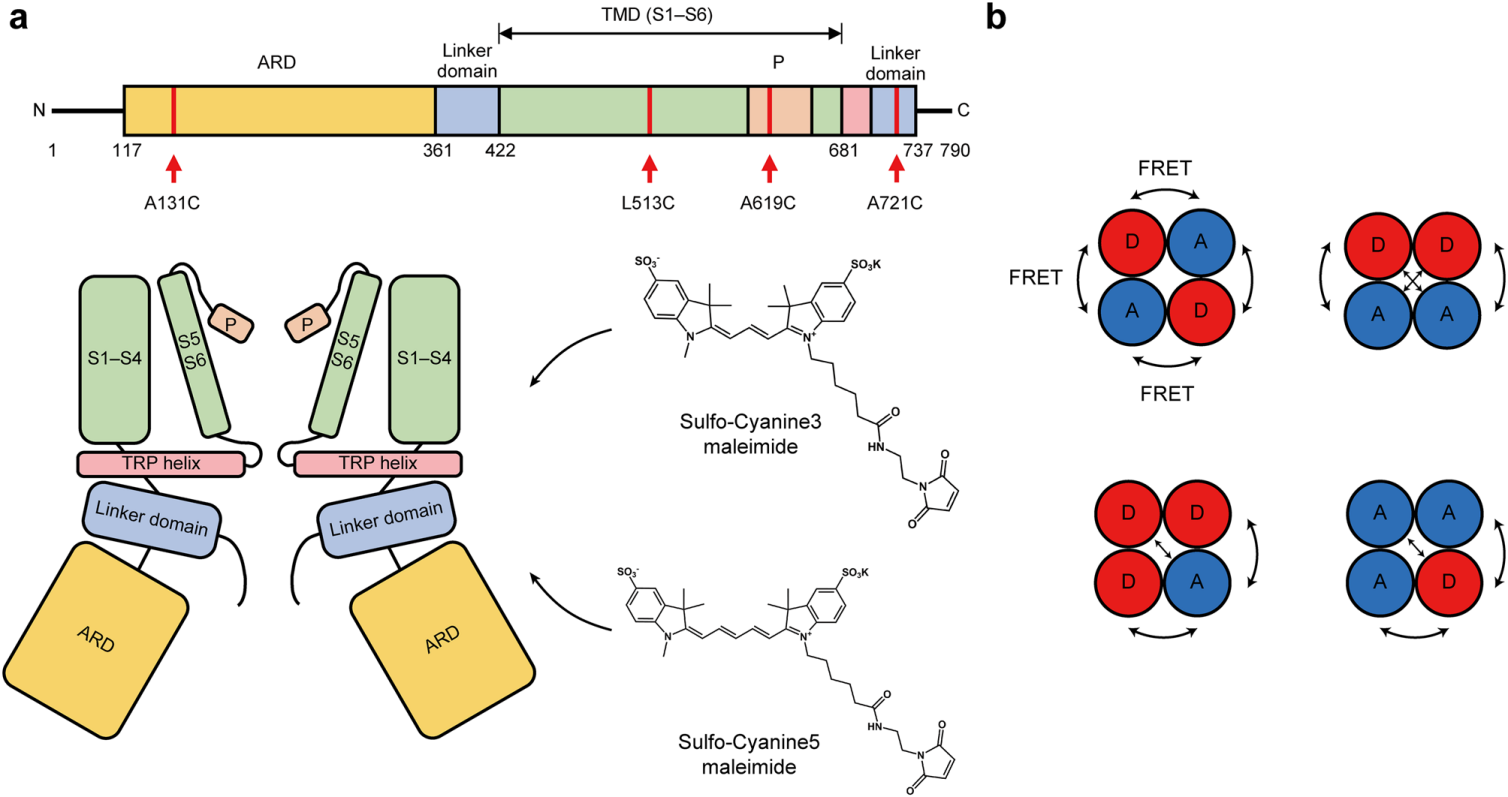
Domain architecture and schematic representation of the fluorescence-labeled TRPV3 and FRET assay. (**a**) Domain architecture of hTRPV3, with locations of cysteines used for dye labeling indicated by red lines. Four residues (A131, L513, A619, and A721 in cysteine-free hTRPV3 are used for fluorescence labeling in the FRET assay. Sulfo-cyanine 3 maleimide and sulfo-cyanine 5 maleimide were used as dye pairs for FRET, and these four residues were replaced by cysteine to covalently link cyanine dyes. (**b**) Possible cases for dye-labeled TRPV3. Both donors and acceptors are randomly labeled into four subunits. FRET occurs when the donor and acceptor are next to or opposite to each other. Black arrows indicate that FRET occurs. *D*, donor; *A*, acceptor.

**Figure 2:**
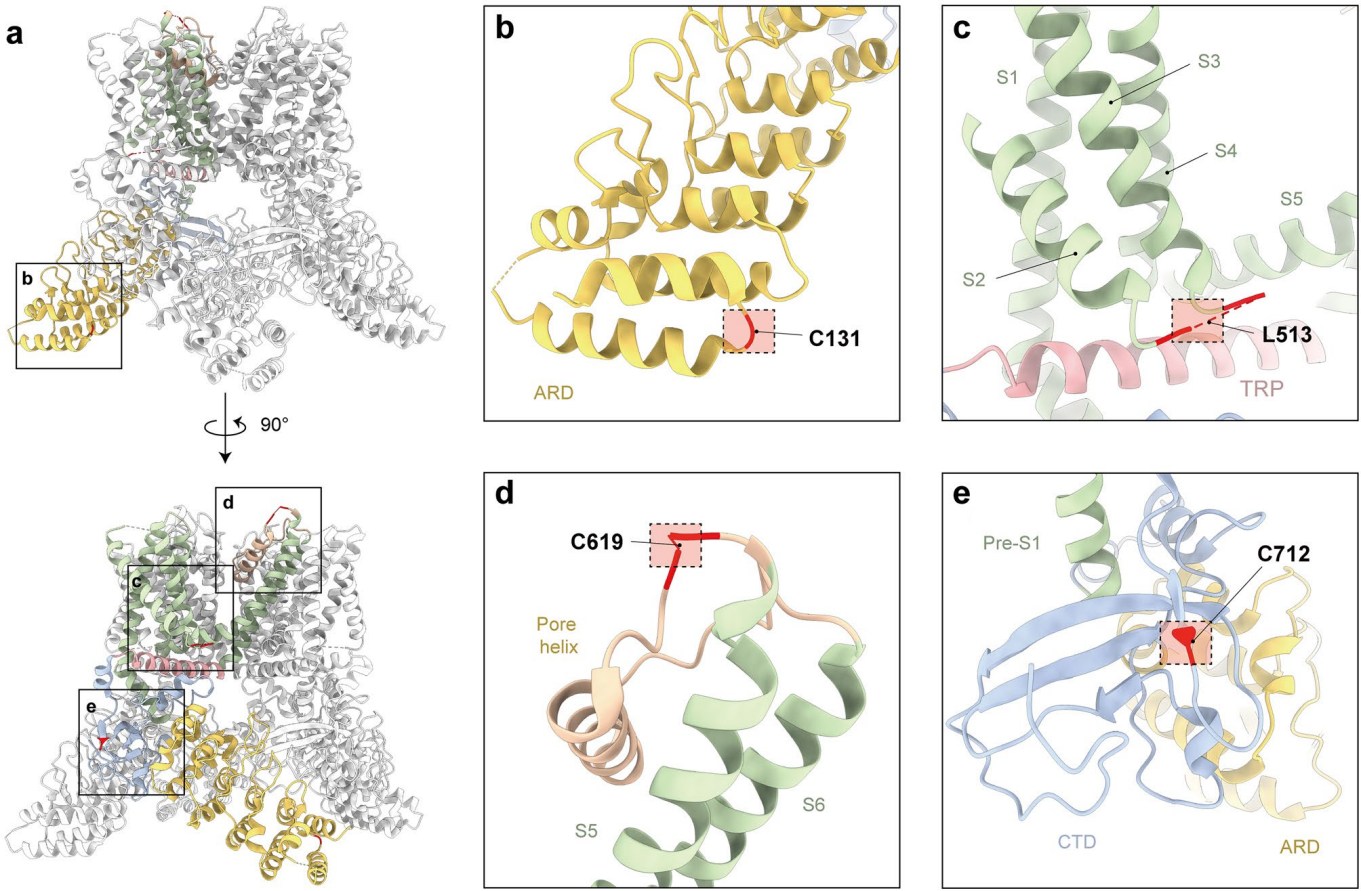
Architecture of TRPV3 constructs for FRET. (**a**) Structural models of hTRPV3 viewed from the side (top) and with 90° rotation (bottom) having residues which are mutated to cysteines colored in red. The structure model of hTRPV3 in the closed state (PDB: 6MHO) is used for representation. Single subunit from tetrameric assembly was colored for better clarity. (**b**) C131 located in ARD. (**c**) L513 located in the S2–S3 linker. (**d**) C619 located in the pore loop between the S5 helix and the pore helix. (**e**) C721 located in the linker domain, especially in the C-terminal domain.

Furthermore, since TRPV3 is homotetrameric and the fluorescence dye will be randomly labeled on each subunit, to observe the change in FRET, the change in distance between the neighboring subunit and the opposite subunit must occur in the same direction: decreasing or increasing in both directions (Fig. 1b). Referring to the domain known to have structural change due to heat in mTRPV3, a total of four residues meeting all these conditions were assigned for dye labeling^7,8^ (Fig. 1a). As a result, three constructs were generated for FRET assay: A131C, A619C, A721C, representing ARD, pore turret domain, and C-terminal linker domain, respectively. In addition, we generated L513C as an exception, as no native cysteine residues represented the transmembrane domain (especially the S2–S3 linker domain). L513 met all the criteria mentioned above, except that it was not a native cysteine (Fig. 2a–e).

In the case of the structures of mTRPV3, wild-type mTRPV3 was not shown to adopt an open conformation after heat stimulation, but the mTRPV3-Y564A mutant showed higher temperature sensitivity, and an open structure was observed after short heat stimulation^7^. Likewise, in hTRPV3, when 2-APB, an agonist of TRPV3, was applied to wild-type hTRPV3, the channel was still in a closed state. However, it was found that the channel easily had an open conformation in the K169A mutant, which is known as a sensitized mutant, by treating it with 2-APB^6,13,20^. Thus, to observe the heat-induced conformational changes in the open state of hTRPV3, we used a TRPV3-K169A mutant that is expected to be easily sensitized by temperature as it was by 2-APB. As a result, four constructs (A131C, L513C, A619C, and A721C) with the K169A mutation were expressed and purified for in vitro FRET assay.

### Conformational changes in hTRPV3 by heat

**Figure 3:**
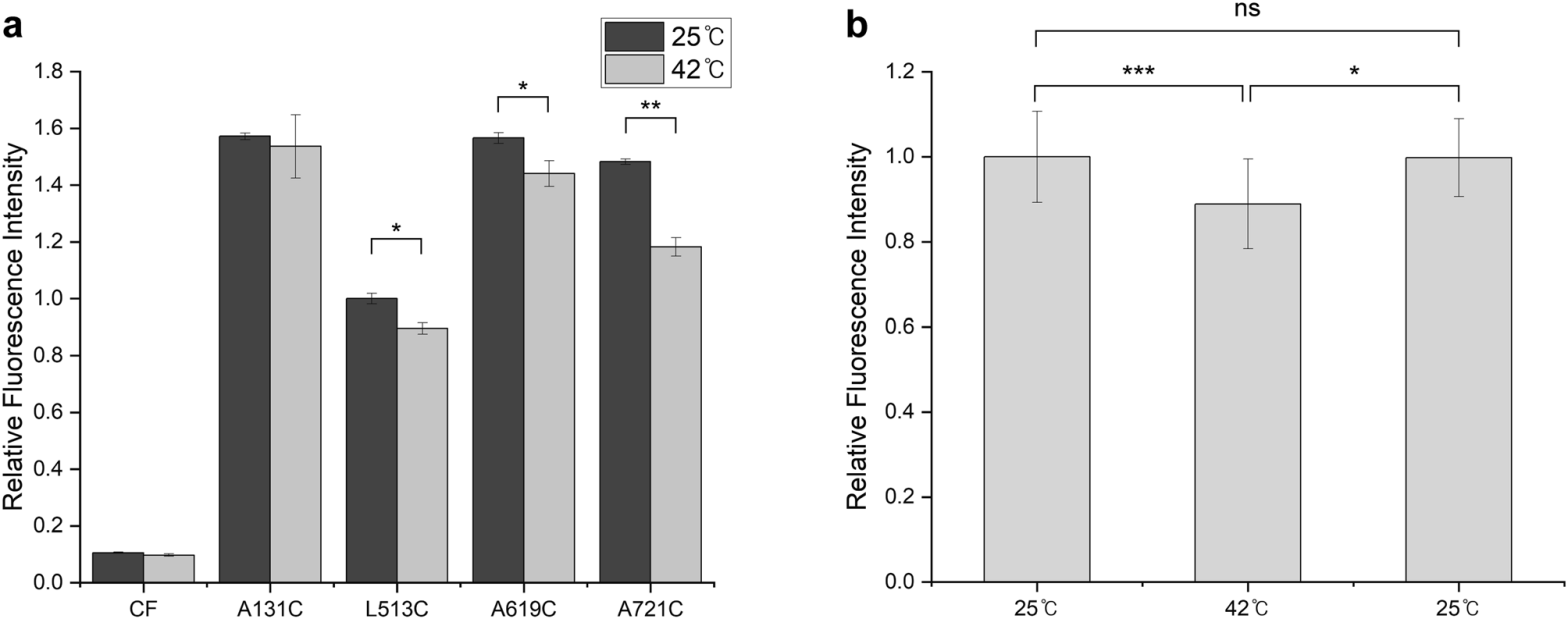
Fluorescence recordings of the heat-stimulated hTRPV3. (**a**) Relative fluorescence intensities measured from four positions: ARD (A131C), S2–S3 linker (L513C), pore turret (A619C), and C-terminal domain (A721C). To normalize fluorescence intensity, the fluorescence intensities of other mutants were normalized to L513C, which had the lowest fluorescence intensity value. The dark bars indicate fluorescence intensity measured at 25 °C after proteins were incubated at room temperature. The light grey bars indicate fluorescence intensity measured at 42 °C after proteins were incubated in heat block at 42 °C. CF indicates cysteine-free hTRPV3, which has no native cysteine in the protein sequence. The CF sample was prepared in the same experimental method including dye labeling, as the other samples. Data are presented as mean ± SEM (n = 3–6). Student’s t-test was used to determine statistical significance; *p < 0.05, **p < 0.01. (**b**) Relative fluorescence intensity measurement at 25 °C, 42 °C, and again at 25 °C using A721C sample. To normalize fluorescence intensity, the fluorescence intensities were normalized to the first 25 °C fluorescence measurement. The fluorescence from the A721C sample was first recorded at 25 °C, then at 42 °C after being incubated in a heat block, and then recorded again at 25 °C after the sample’s temperature was slowly lowered at room temperature. Data are presented as mean ± SEM (n = 6). Student’s t-test was used to determine statistical significance; *p < 0.05, **p < 0.01, ***p < 0.001.

To gain information on temperature-dependent structural dynamics in hTRPV3, we performed FRET with different hTRPV3 constructs (CF, A131C, L513C, A619C, and A721C). We attached Cy3 and Cy5 to cysteine residues in each construct and monitored conformational changes by measuring fluorescence intensities at two different temperature conditions, i.e., at 25 °C and 42 °C (Fig. 1a). As a result, changes in fluorescence intensity were observed, which were related to the structural changes followed by channel activation, except for the CF construct which was used for the negative control^21^. Fluorescence intensities of the samples showed an overall decrease during heat-induced hTRPV3 activation (Fig. 3a). All constructs exhibited different degrees of FRET decrease. Among them, A131C showed almost no difference in FRET, indicating that the structural change did not occur by heat stimulation at the ARD. Compared to A131C, there were significant changes in FRET signals in other domains. The most substantial change was observed in A721C, which represents the linker domain, especially in the C-terminal domain, followed by A619C (pore domain) and L513C (S2–S3 linker). In mTRPV3, the C-terminus, linker domain, and S2–S3 linker undergo notable structural changes, but the ARD undergoes only a rigid body motion during heat-induced activation. Many other studies have identified that the C-terminus and pore loop play important roles in the thermal activation of thermoTRP channels^7,8,21–25^. These FRET changes indicate that the largest structural change of hTRPV3 upon heat activation occurs in the C-terminal domain, located right forward of the C-terminus in sequence, and a slight structural change also occurs in the pore domain and the S2–S3 linker.

In interpreting the FRET results, it might be possible that the proteins were damaged by heat stimulation and lost their tetrameric assembly, which might also lead to a decrease in FRET signals. To ensure that the protein remains intact after heat application, we lowered the temperature of the sample to 25 °C which had been heated to 42 °C, and measured fluorescence again. As a result, the fluorescence intensity was recovered to a similar level to the value before the application of heat (Fig. 3b). Furthermore, fluorescence-detection size-exclusion chromatography (FSEC) results also confirmed that proteins were not severely damaged and did not lose their oligomeric assembly after thermal stimulation. We performed FSEC with heat-treated samples for each construct and compared their chromatograms to the chromatogram with a heat-untreated sample. As a result, all heat-treated and heat-untreated samples showed similar patterns in their chromatograms, and hTRPV3s were eluted at the retention time consistent with the tetrameric size (Supplementary Fig. S1). These two additional experiments verified that FRET changes were due to the structural changes of the proteins, not to aggregated or disassembled proteins upon heat application.

### Conformational changes in hTRPV3 by carvacrol

**Figure 4:**
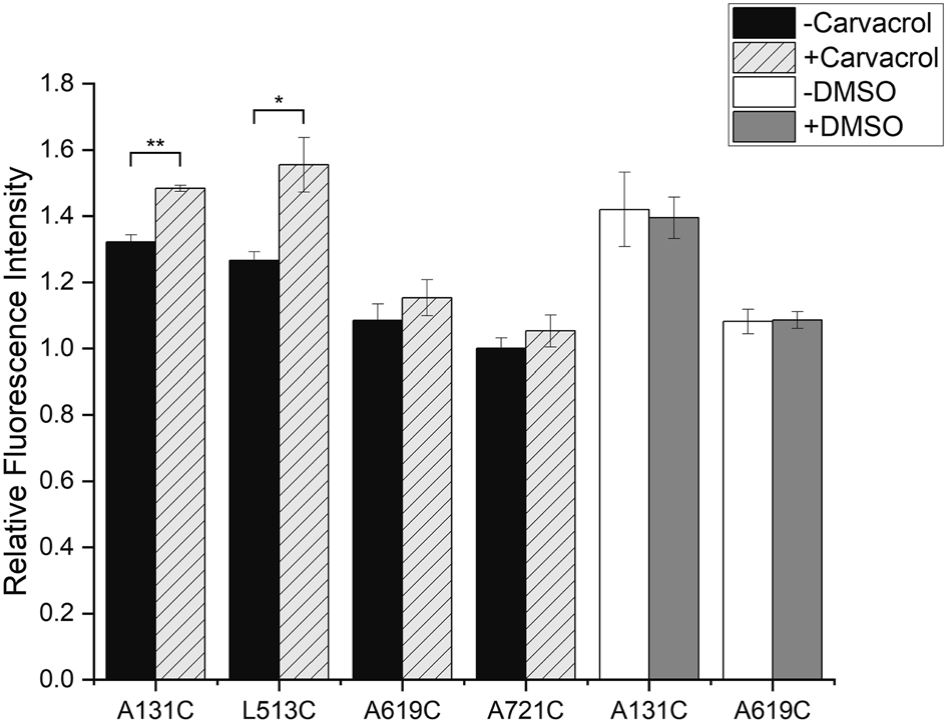
Fluorescence recordings of the carvacrol-stimulated hTRPV3. Relative fluorescence intensity recorded from four positions: ARD (A131C), S2–S3 linker (L513C), pore turret (A619C), and C-terminal domain (A721C). To normalize fluorescence intensity, the fluorescence intensities of other mutants were normalized to A721C, which has the lowest fluorescence intensity value. The black bar and white bar indicate the fluorescence intensity value of the samples that have not been treated with anything including carvacrol or DMSO. The fluorescence intensity values of the samples treated with carvacrol were showed in a comb pattern bar, and samples incubated with DMSO were showed in grey bar. Data are presented as mean ± SEM (n = 3–6). Student’s t-test was used to determine statistical significance; *p < 0.05, **p < 0.01.

Since the activation mechanism of TRPV3 by agonists other than 2-APB remains unknown, we used carvacrol, which is a plant-derived specific agonist of TRPV3, and tried to examine the carvacrol-induced conformational changes of TRPV3 using FRET assay and compared those to the results of the heat-induced conformational changes^11^. We performed FRET assays with different hTRPV3 constructs (A131C, L513C, A619C, A721C) with or without carvacrol treatment. Interestingly, unlike in the case of heat-induced activation, increased FRET signals were observed during carvacrol activation (Fig. 4). A131C (ARD) and L513C (S2–S3 linker) generated substantial FRET increases, but A721C showed a considerably low change in FRET, and these results were completely different from the results after heat activation (Figs. 3a and 4). In interpreting these results, one might claim that the solvent used for dissolving carvacrol was responsible for the different results. To confirm that the carvacrol was the reason for the FRET signal difference, we performed the same FRET experiments with some constructs (A131C and A619C) with or without dimethyl sulfoxide (DMSO, no carvacrol dissolved). As a result, the fluorescence intensities were almost the same regardless of the DMSO treatment suggesting that DMSO itself is independent of FRET change, and fluorescence changes in TRPV3 imply structural changes by carvacrol binding. In fact, a previous study suggested that the S2–S3 linker is forming a binding pocket for carvacrol by using molecular docking^15^. In the TRPV3 structure, carvacrol seems to interact with residues in the S2–S3 linker and fits well on the surface of TRPV3^15^. Our results support that the S2–S3 linker has a notable structural change by carvacrol activation, and the S2–S3 linker of hTRPV3 might be crucial to carvacrol binding and activation. Moreover, our results also indicate that carvacrol-induced activation is distinct from heat-induced activation, suggesting that structural changes might occur in a different mechanism.

## Discussion

In this work, we devised an experiment to find out the domain of a protein in which the structural change occurs by using FRET. In previous research, a fluorescence dye pair was attached to the cysteine of the pore turret of thermoTRP in a cell, and the temperature-dependent fluorescence change was observed through FRET assay^21^. Structural changes of proteins in the native environment could be observed by in vivo FRET. However, it is difficult to specifically attach the fluorescence dyes to the target proteins in the in vivo FRET assay, and the influence of other factors present in the cell cannot be excluded. We expected that these issues could be overcome by performing in vitro FRET. We designed an experiment to perform a FRET assay using purified membrane protein to record fluorescence by attaching the fluorescent dye only to TRPV3 itself and observing structural changes of the protein by only one variable, such as heat and ligand.

Caution was warranted while introducing the K169A mutation to all constructs because it might not represent the apo state of TRPV3. However, we inevitably introduced the K169A mutation for better heat-induced structural changes because no changes were observed when a single heat pulse was exerted on wild-type constructs (Supplementary Fig. S5a). Our results clearly suggest that thermal stimuli did induce structural changes to the constructs with the K169A mutations as we can infer from the FRET changes before and after heat activation (Fig. 3a), indicating heat-induced structural changes. If the K169A mutation made the constructs constitutively and fully active, no FRET changes would have been detected. Moreover, control experiments with/without treatment of 2-APB showed no FRET changes on the A721C construct regardless of the K169A mutation (Supplementary Fig. S5b).

When hTRPV3 was exposed to high temperature, remarkable structural changes occurred in the C-terminal domain, pore turret, and S2–S3 linker in order of the degree of change, but no change in distance was observed in the ARD. Comparing the closed-state and open-state conformations of mTRPV3 by temperature, there was no change in the distance between subunits in the ARD, and the largest change in distance between the two conformations in the C-terminal domain, followed by the pore turret and S2–S3 linker^7^. We figured out that hTRPV3 will change its structure in the same way that mTRPV3 adopts with changes in the C-terminal domain, pore domain, and S2–S3 linker at high temperatures because they have high sequence identities^7,8^. Also, as in mTRPV3, when hTRPV3 is stimulated by heat, no structural change occurs between subunits, although rigid body motion can occur in ARD, unlike in other domains^7,8^. In particular, because the largest structural change occurred in the C-terminal domain close to the C-terminus, it is likely that the C-terminus still plays a major role in the temperature-dependent activation of human thermoTRP channels. However, the distance between subunits in the S2–S3 linker and pore turret decreased when the temperature increased in the mTRPV3, but increased in the hTRPV3^7^. This might be a difference between mouse and human TRPV3, but a more definite reason for this will be revealed through further structural studies.

When carvacrol was applied to hTRPV3, we observed structural changes in the S2–S3 linker and ARD, indicating that they might be part of the carvacrol activation pathway in hTRPV3. Comparing the TRPV3-K169A structures before and after treatment with 2-APB, there were relatively no noticeable changes in domains other than the pore turret among the four domains, but with carvacrol treatment, changes were observed in other domains, not in the pore turret^13^. According to a recent study, carvacrol is predicted to bind to a pocket containing the S2–S3 linker, which is a unique site completely different from the 2-APB binding site of TRPV3. Therefore, it is expected that TRPV3 will have a different structure when activated by carvacrol^15^. Our study implies that when hTRPV3 is stimulated by the natural compound carvacrol, the structural change occurs at the S2–S3 linker, which is a putative binding site. This change might be transmitted to other domains, followed by a change in ARD. It is also worth noting that S2–S3 region would not be the only region that responds to the carvacrol binding. According to the recent electrophysiology data of mTRPV3, S4–S5 region was responsible for channel activation via menthol or camphor^26^. Considering that these compounds also belong to monoterpenes, like carvacrol, and that there are high similarities between hTRPV3 and mTRPV3, it is highly plausible that the S4–S5 region will be involved in the structural changes after activation by carvacrol.

We propose that thermoTRPs respond to heat and ligand stimuli with different structural mechanisms^21^. By comparing activation through heat stimulation and ligand stimulation, we observed that conformational changes occur in different domains, suggesting that heat and ligand activate the protein in different ways. In temperature-dependent activation, the largest change occurred in the C-terminal domain, but no change was observed in this domain in carvacrol-dependent activation, and the opposite result was found in ARD. Through FRET assay, we visually identified the regions that respond to temperature change and carvacrol in hTRPV3, which helps to understand how the human thermoTRP channel responds to temperature and ligands. Obtaining additional cryo-EM structures of heat- and/or carvacrol-activated hTRPV3 would enable us to bridge the current gaps between our findings and the authentic nature of hTRPV3, thereby contributing to a more complete understanding of its activation mechanism and facilitating the development of effective therapeutic interventions targeting this protein.

Since TRPV3 plays an essential role in pain generation, early research on TRPV3 mainly focused on pain and thermal sensation. Further studies on physiological functions have shown that TRPV3 is associated with various diseases, making it a potential drug target. However, the potentially different expression profile of TRPV3 between humans and rodents may complicate preclinical translation into humans^27^. Therefore, understanding the biology of the hTRPV3 channel is crucial, and in this sense, our results provide insights into understanding the molecular mechanisms of hTRPV3, which are necessary for drug development.

## Methods

### Constructs

The full-length cysteine-free human TRPV3 was subcloned into a pFastBac vector with a FLAG affinity tag. This construct has all 13 cysteines substituted by other amino acids: C75A, C80A, C131A, C146A, C171A, C271A, C446L, C496L, C550F, C612A, C619A, C721A, and C731A^24,28^. For the FRET assay, single Cys mutants of cysteine-free TRPV3 were designed, which contain one Cys in each of the different domains in order to observe conformational changes by domain: A131C for ARD, L513C for S2–S3 linker, A619C for pore turret, and A721C for C-terminal domain, respectively. All of these mutants have the K169A mutation to observe the structural changes in the open conformation, and were produced by a site-directed mutagenesis procedure.

### Protein expression and purification

TRPV3 constructs were expressed in Sf9 cells using baculovirus. The baculovirus was produced according to the manufacturer’s protocol (Invitrogen, Bac-to-Bac), then applied to Sf9 cells and cultured at 27 °C for ~ 72 h. After transduction, the cells were collected by centrifugation and resuspended in a buffer containing 50 mM Tris pH 8.0, 150 mM NaCl, 2 µg mL^−1^ leupeptin, 2 µM pepstatin, 2 µM aprotinin, and 1 mM phenylmethylsulfonyl fluoride. Cells were subsequently solubilized for 2 h in a buffer containing 40 mM n-dodecyl β-D-maltoside (DDM, Goldbio) and 4 mM cholesteryl hemisuccinate tris salt (CHS, Anatrace) with rotating at 4 °C for membrane protein extraction. After solubilization, the cell lysate was centrifuged at 100,000* g* for 1 h to remove unsolubilized material. The supernatant was added to anti-FLAG resin and incubated for 3 h at 4 °C with rotation. After sample binding to the resin, the resin was washed with 10 column volumes of wash buffer (50 mM Tris pH 8.0, 150 mM NaCl, 1 mM DDM, 0.1 mM CHS), and the protein was eluted with elution buffer [50 mM Tris pH 8.0, 150 mM NaCl, 1 mM DDM, 0.1 mM CHS, 0.1 mg ml^−1^ 3:1:1 1-palmitoyl-2-oleoyl-sn-glycero-3-phosphocholine (POPC), 1-palmitoyl-2-oleoyl-sn-glycero-3-phosphoethanolamine (POPE), 1-palmitoyl-2-oleoyl-sn-glycero-3-phospho-(1'-rac-glycerol) (POPG), 0.5 mg ml^−1^ FLAG peptide] (Supplementary Fig. S2a). After the FLAG-affinity chromatography, a small portion of the elution fractions was directly injected into the Superose 6 Increase 10/300 GL column (Cytiva) to confirm that the protein showed tetrameric assembly (Supplementary Fig. S3). For the preparation of the samples for dye labeling, 5 mM dithiothreitol (DTT) was added to the eluted protein and incubated for 30 min on ice to reduce cysteine. DTT and FLAG peptides were removed, and protein was concentrated to 1 mg ml^−1^ using 100 kDa MWCO centrifugal filter (Millipore Amicon).

### Protein labeling

To label the proteins with dye, a 1:1 mixture of sulfo-Cy3 maleimide (Lumiprobe) and sulfo-Cy5 maleimide (Lumiprobe) was added to the proteins at a protein:fluorophore molar ratio of 1:10. The labeling reaction was performed at 4 °C for 2 h. The labeled proteins were then diluted and added to anti-FLAG resin, and the excess free dyes were effectively removed by extensive washing^29^. The proteins were eluted by elution buffer and concentrated to 0.3–0.4 mg ml^−1^ (Supplementary Fig. S2b).

### FRET assay

FRET was detected using a microplate reader Synergy TM H1 (BioTek). The plate was read with an excitation wavelength of 548 nm from a Xenon lamp, and fluorescence emission was recorded at 662 nm. For the preparation of FRET samples to observe changes by heat, the fluorescence intensities of proteins were first measured at 25 °C, and then measured again using the same samples in the instrument pre-heated to 42 °C in order to observe changes in fluorescence intensity from temperature-induced structural changes in the protein. Before measuring the protein samples at 42 °C, the proteins were subjected to heat stimulation by using a heating block. The samples were incubated for 1–2 min, which is considered sufficient time for the protein of sensitized phenotype activation, in a heating block equilibrated at 42 °C^7,13^. For the preparation of FRET samples to observe changes induced by carvacrol, the fluorescence intensities of proteins were first measured at room temperature. Then, 1 mM carvacrol was added to the samples, and the fluorescence intensities were measured again at room temperature using the same samples supplemented with carvacrol. Carvacrol (Sigma-Aldrich) was prepared in dimethyl sulfoxide. The proteins were treated with carvacrol for 1–2 min, as in the heat samples. To compare conformational changes at different positions under different conditions, fluorescence intensities were measured in repeated experiments and averaged under various conditions^21^. For the preparation of FRET samples to observe changes induced by 2-APB, all the experimental procedures were identical to those with carvacrol except that 2-APB was used instead of carvacrol.

## Supplementary Information

Supplementary Information.

## Data Availability

All data generated or analyzed during this study are included in this article.
