# Supplementary material for: FRET analysis of the temperature-induced structural changes in human TRPV3
Source: Sci Rep. 2023 Jun 21;13:10108. doi: 10.1038/s41598-023-36885-9 (PMC10284872; doi:10.1038/s41598-023-36885-9)
Supplement: Supplementary file 1 — Supplementary Information. [file 41598_2023_36885_MOESM1_ESM.docx]

**FRET analysis of the temperature-induced structural changes in human TRPV3**

Jinyoung Kim^1,*^, Jongdae Won^1,*^, Dong Kyu Chung^1^, and Hyung Ho Lee^1,†^

^1^Department of Chemistry, College of Natural Sciences, Seoul National University, Seoul 08826, Korea

*These authors contributed equally to this work

^†^Corresponding author:

Professor Hyung Ho Lee, Department of Chemistry, College of Natural Sciences, Seoul National University, Seoul 08826, Korea;

Correspondence to hyungholee@snu.ac.kr

*Keywords*: ThermoTRP, TRPV3, Fluorescence resonance energy transfer, Conformational change, Thermosensation

**Supplementary Figures**


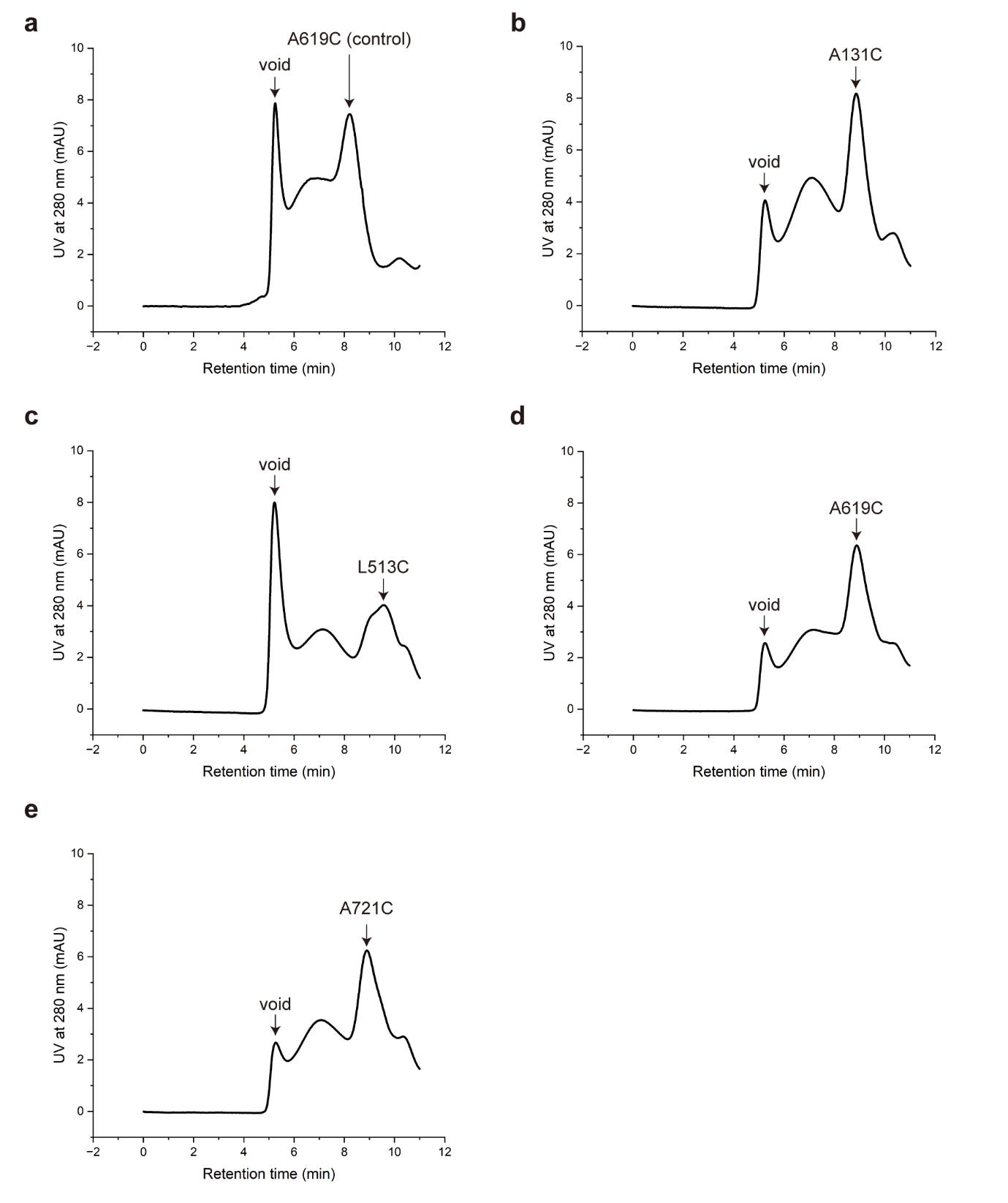


**Supplementary Figure S1. Size-exclusion chromatography of human TRPV3 after FRET.** (a)–(e) Size-exclusion chromatography of hTRPV3 FRET samples using Superose 6 Increase 5/150 GL column. (a) Size-exclusion chromatography of TRPV3-A619C without heating to identify the retention time of the TRPV3 tetramer. (b)–(e) Size-exclusion chromatography of TRPV3 FRET samples after heat stimulation. All the samples were eluted at a retention time of TRPV3 tetramer; A131C (b), L513C (c), A619C (d), A721C (e).


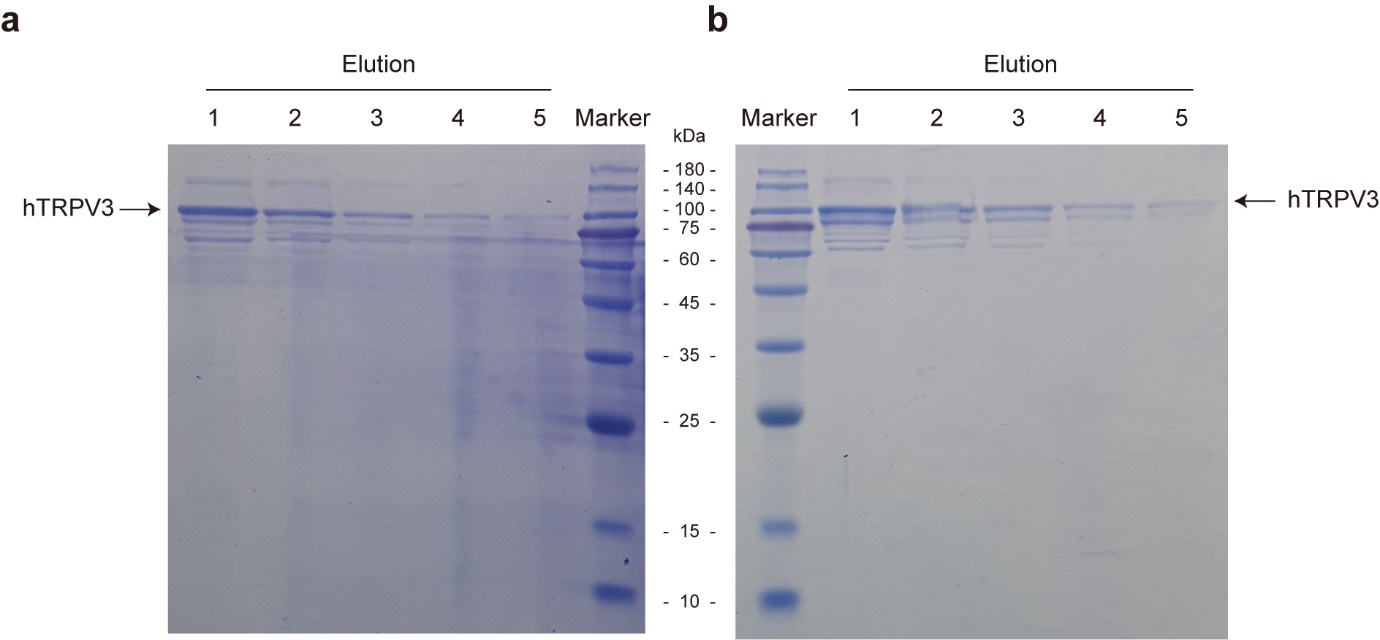


**Supplementary Figure S2. Human TRPV3 purified by affinity chromatography.** (a), (b) SDS-PAGE analysis of hTRPV3 1^st^ FLAG affinity (a) and 2^nd^ FLAG affinity after dye labeling (b). SDS-PAGE gels were visualized by using Coomassie blue. Lanes 1-5 are elution fractions of FLAG affinity chromatography.


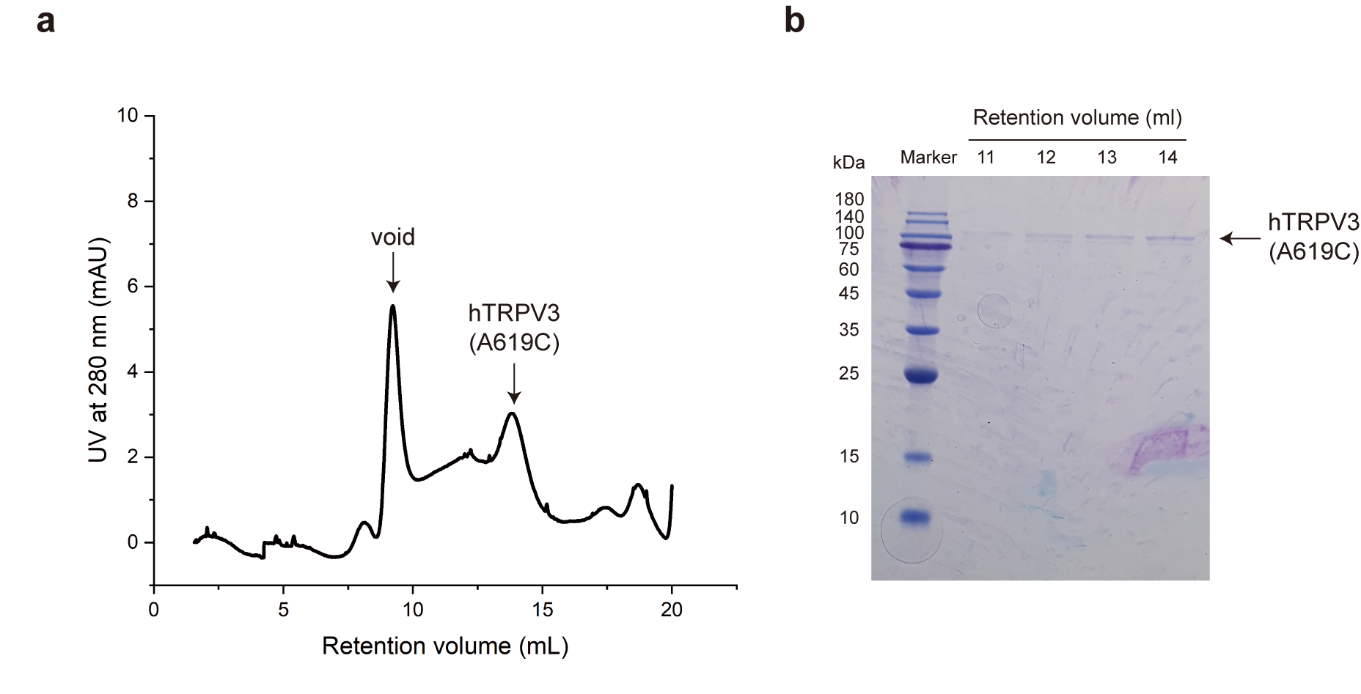


**Supplementary Figure S3. Human TRPV3 purified by size-exclusion chromatography.** (a) Size-exclusion chromatography of hTRPV3-A619C using Superpose 6 Increase 10/300 GL column. (b) The peak fraction of TRPV3-A619C was shown on SDS-PAGE stained with Coomassie blue.


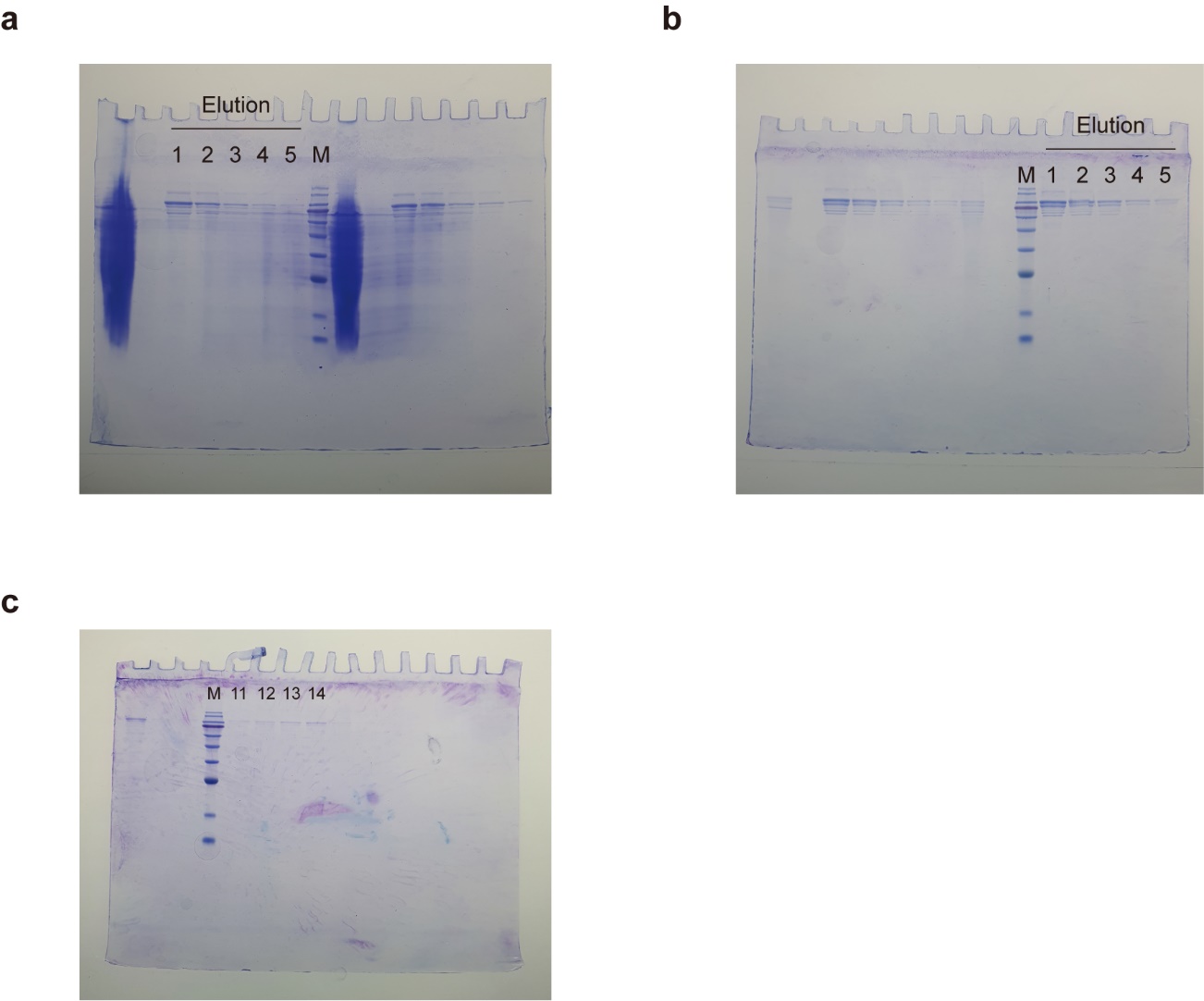


**Supplementary Figure S4. Uncropped picture of SDS-PAGE.** (a) Uncropped picture of SDS-PAGE in Figure S2(a). (b) Uncropped picture of SDS-PAGE in Figure S2(b). (c) Uncropped picture of SDS-PAGE in Figure S3(b). Lanes are labeled accordingly as in Figure S2(a), (b) and S3(b), respectively. M, Marker.


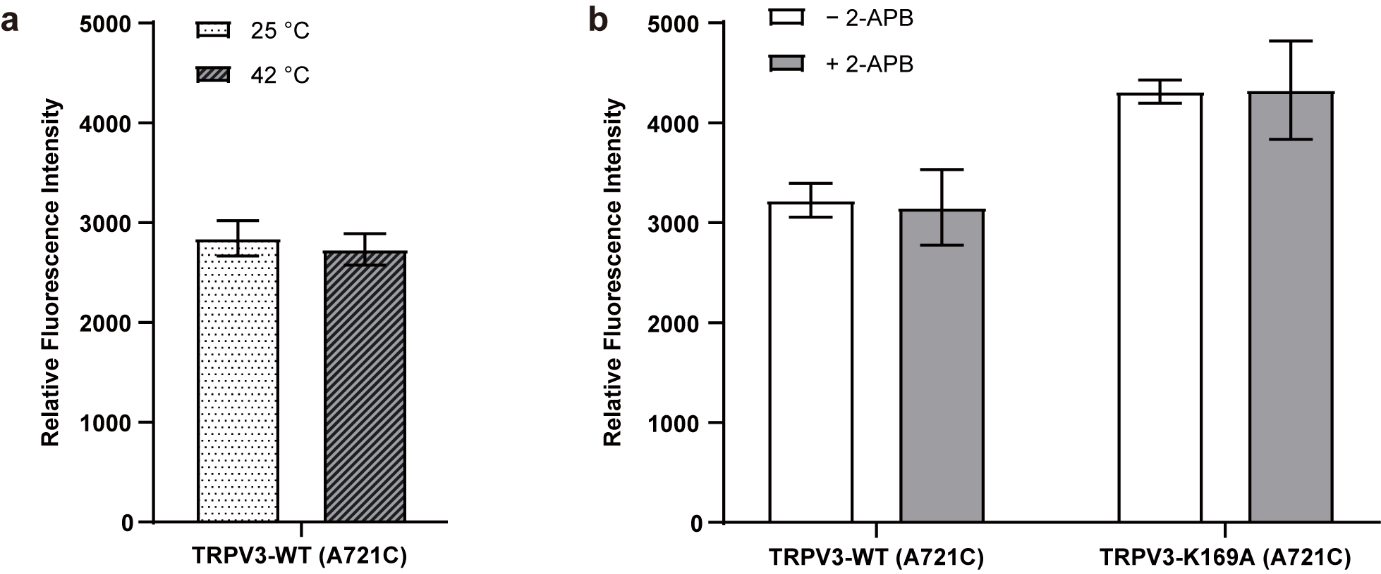


**Supplementary Figure S5. Fluorescence recordings of the heat- or 2-APB-stimulated TRPV3-WT.** (**a**) Relative fluorescence intensities measured from the TRPV3-WT (A721C) construct before and after thermal stimuli. The protein sample was incubated at 42℃ prior to the fluorescence measurement at 42℃. (**b**) Relative fluorescence intensities measured from the TRPV3-WT (A721C) and the TRPV3-K169A (A721C) constructs before and after the 2-APB treatment.

# Supplementary Table

**Supplementary Figure S1. Classification of native cysteine residues in human TRPV3.** Residues in bold (C131, C619, C721) represent the selected cysteine residues for FRET experiments in this study.

| **Native cysteine residues** | Representative structural domain |
| --- | --- |
| C75, C80 | N-terminal region (unresolved) |
| **C131**, C146, C171, C271 | Ankyrin repeat domain |
| C446, C496, C550 | Transmembrane domain |
| C612, **C619** | Pore domain |
| **C721**, C731 | C-terminal linker domain |
